# Transient Gastric Irritation in the Neonatal Rats Leads to Changes in Hypothalamic CRF Expression, Depression- and Anxiety-Like Behavior as Adults

**DOI:** 10.1371/journal.pone.0019498

**Published:** 2011-05-12

**Authors:** Liansheng Liu, Qian Li, Robert Sapolsky, Min Liao, Kshama Mehta, Aditi Bhargava, Pankaj J. Pasricha

**Affiliations:** 1 Division of Gastroenterology and Hepatology, Stanford University Medical Center, Stanford, California, United States of America; 2 Department of Pharmacology and Toxicology, University of Kansas, Kansas City, Kansas, United States of America; 3 Department of Biology, School of Humanities and Sciences, Stanford University, Stanford, California, United States of America; 4 Department of Surgery, University of California San Francisco, San Francisco, United States of America; Chiba University Center for Forensic Mental Health, Japan

## Abstract

**Aims:**

A disturbance of the brain-gut axis is a prominent feature in functional bowel disorders (such as irritable bowel syndrome and functional dyspepsia) and psychological abnormalities are often implicated in their pathogenesis. We hypothesized that psychological morbidity in these conditions may result from gastrointestinal problems, rather than causing them.

**Methods:**

Functional dyspepsia was induced by neonatal gastric irritation in male rats. 10-day old male Sprague-Dawley rats received 0.1% iodoacetamide (IA) or vehicle by oral gavage for 6 days. At 8–10 weeks of age, rats were tested with sucrose preference and forced-swimming tests to examine depression-like behavior. Elevated plus maze, open field and light-dark box tests were used to test anxiety-like behaviors. ACTH and corticosterone responses to a minor stressor, saline injection, and hypothalamic CRF expression were also measured.

**Results:**

Behavioral tests revealed changes of anxiety- and depression-like behaviors in IA-treated, but not control rats. As compared with controls, hypothalamic and amygdaloid CRF immunoreactivity, basal levels of plasma corticosterone and stress-induced ACTH were significantly higher in IA-treated rats. Gastric sensory ablation with resiniferatoxin had no effect on behaviors but treatment with CRF type 1 receptor antagonist, antalarmin, reversed the depression-like behavior in IA-treated rats

**Conclusions:**

The present results suggest that transient gastric irritation in the neonatal period can induce a long lasting increase in depression- and anxiety-like behaviors, increased expression of CRF in the hypothalamus, and an increased sensitivity of HPA axis to stress. The depression-like behavior may be mediated by the CRF1 receptor. These findings have significant implications for the pathogenesis of psychological co-morbidity in patients with functional bowel disorders.

## Introduction

Functional dyspepsia (FD), defined as persistent or recurrent pain in the upper abdomen presumably of gastroduodenal origin but without any structural etiology to explain the symptoms, is a common clinical gastrointestinal disorder that affects 15–20% of the population [1]–[4]. A number of mechanisms have been suggested to explain the symptoms, centered around altered neuromuscular and/or sensory activity of the stomach or duodenum [2]. A major theme in this field is that psychological/psychiatric problems have a pathogenic role, based both on the observation that patients with FD are more anxious and depressed than healthy controls[1], [3]–[5], as well as research linking stress and depression to altered gastrointestinal sensory and motor function [6]–[10]. In this study, we hypothesized that primary visceral disturbances in early life can result in persistent behavioral and emotional abnormalities. This hypothesis is based on our previous published work showing that the neonatal period is a particularly vulnerable period for the viscera. Transient inflammation or injury of either the stomach or colon result in long-lasting hypersensitivity and motor abnormalities that persist despite complete resolution of the initial insult [11]–[14].

To test this hypothesis, we used a previously validated model of FD in rats, induced by a mild gastric irritation, resulting in chronic gastric hypersensitivity and motor dysfunction in adulthood [11]. Our results suggest that transient gastric irritation in the neonatal period can induce long-lasting increases in depression-,and anxiety-like behaviors and increase in the HPA axis sensitivity to stress that is associated with increase the expression of corticotropin-releasing factor (CRF) in the paraventricular nucleus (PVN) of the hypothalamus.

## Materials and Methods

### Animals

The experimental protocols, care and handling of animals used in this study (Protocol ID 18206) were approved by the Institutional Animal Care and Use Committee at Stanford University, in accordance with the guidelines of the International Association for the Study of Pain. Male Sprague-Dawley rats were used in all the experiments (Harlan, Indianapolis, IN). 6-day old pups with dams were purchased from Harlan, Indianapolis, IN. The treatment was started when the pups were 10 day old. The housing conditions of the mother/pups were as follows: temperature 68–71F°; 12/12 light cycle; humidity 43–53%; sanichip for bedding; enrichment for 1 mother with 10 pups. They had *ad libitum* access to food and water. Pups were weaned at age of 3 weeks and were housed 3–4 pups per cage. At age 6 weeks, the rats were housed two per cage.

### Functional Dyspepsia (FD) Model

The “functional dyspepsia” model has been previously described by us and consists of neonatal administration of a mild irritant, which results in transient superficial sloughing of the gastric epithelium [11]. As adults, the stomach is morphologically and histologically normal, as is gastric emptying. However, there is significant hypersensitivity to gastric distention manifested both by behavioral changes and gastric splanchnic activity as well as impaired accommodation [11]. Ten-day old rat pups received 0.2 ml of 0.1% iodoacetamide (IA) in 2% sucrose daily for 6 days by oral gavage. Control rat pups received 0.2 ml of 2% sucrose. The rats were then allowed to grow to 8–10 weeks of age, whereupon tests described below were conducted.

### Behavioral testing

Rats were handled for 3 days prior to the behavioural test day. On the test day, rats were brought to the test room for at least one hour before the test. Behavioural tests were conducted in sequential order of Sucrose Preference Test (SPT), Open Field Test (OFT), Elevated Plus Maze (EPMT), Light Dark Box (LDB) and Forced Swimming Test (FST) with one to two day intervals between the tests.

#### Sucrose Preference Test (SPT) [15]–[17]

This consisted of a 48 hour training session and a 1 hour test session conducted 24 hours after the training session. In the training session, singly housed rats were trained to drink sugar water in a cage containing two bottles, one bottle containing a 1% sucrose solution and another bottle containing tap water for 48 hours. The bottles were placed to the left and right side of the feeding compartment, respectively and were switched every 12 hours to prevent possible effects of side preference in drinking behavior. After the training session, only tap water was provided for 6 hours. Then food and water were withheld from rats for 18 hours. Subsequently, in the test session, rats were provided access to two bottles with 1% sucrose solution and water, respectively, for one hour. Sucrose preference was analyzed according to the formula below:

Sucrose preference (SP)  =  [sucrose intake (g)/(sucrose intake (g) + water intake (g))]×100

The proportion of rats in each group with an SP value of ≥75% was then counted and compared using the Chi-square test.

#### Forced Swimming Test (FST) [18]–[20]

A clear Plexiglas cylinder (65 cm tall × 25 cm diameter) was filled to 48 cm with 25°C water. On the training day, the rats were placed in the cylinder for 10 minutes and then removed from the water. 24 hours later, rats were retested for 5 minutes under identical conditions and their behavior recorded on videotape. Videotapes were scored by a blind examiner using a time-sampling technique: the behavior at the end of each 5-second period was categorized as one of the following: 1) immobility: the rat remained floating in the water without struggling and made only those movements necessary to keep its head above water; 2) swimming: the rat displayed active swimming motions, more than necessary to merely maintain its head above water, e.g. moving around in the cylinder; 3) climbing: the rat displayed active movements with its forepaws in and out of the water, usually directed against the walls.

#### Elevated Plus Maze (EPM) Test [21]

The EPM apparatus, made of black polypropylene, consisted of two opposite closed and two opposite open arms (10 cm width) with 30 cm high walls and a center area (10 × 10 cm), elevated 50 cm above the floor. Rats were placed individually in the center of the maze facing an open arm and were allowed 5 minutes of free exploration. The movements of the animals during the 5-minute test period were tracked by a video camera positioned above the center of the maze and analyzed using Ethovision (Noldus Information Technology) video tracking system to evaluate the time spent the open arms, percentage moved in open arm and total distance moved during the 5-minute test time.

#### Open Field Test (OFT) [22], [23]

The open field apparatus consisted of an arena (100 cm × 100 cm × 40 cm) made of black plastic, which is dimly illuminated (corner: 6 lx; center: 12 lx). A rat is gently placed into the corner of the field, and allowed to explore the arena for 30 minutes. Movements are recorded by a video camera mounted above the arena, and analyzed using the video tracking system. The number of entries into the center, the percentage time in the center, the total distance traveled and the proportion traveled in the center during the test period were measured.

#### Light Dark Box (LDB) Test [24], [25]

The apparatus for this test consisted of two equally sized compartments (h×w×l: 24×25×33 cm ) connected by an 8×8 cm opening. One compartment was painted black and covered with a black lid (dark box). The other compartment was opaque and remained uncovered during the test (light box). An opaque black Plexiglas with a door separated the two boxes. Inside the light box was illuminated by a 30 lux light. Inside the dark box there was no appreciable illumination (<2 lux). On test days, rats were moved to the test room 60 minutes prior to testing. Assessments were performed between 10 a.m. and 3 p.m. To start the test, the rat was placed in the center of the light box facing away from the door to the dark box and allowed to freely explore the apparatus for 5 min. The movements of the animals during the 5 minute test period were tracked by a video camera positioned above the center of the light dark box and analyzed using the video tracking system (Noldus Information Technology).The data of behavior analysis included the percentage of time spent in the light box and dark box and the number of chamber transitions, defined as at least half of the animal's body from one chamber to the next.

### Hypothalamic Pituitary Axis (HPA) response to minor stressor, saline injection

IA-treated and control rats were divided into two groups and were housed two per cage. The rats in the same cage were assigned to the same group. In one group, rats were subjected to a minor stressor, subcutaneous injection of saline (1 ml/kg,) and decapitated 15 minutes later. In another group, rats were decapitated without interruption. Trunk blood was collected from rats in both groups for hormonal assays. Plasma ACTH and corticosterone concentrations were determined by radioimmunoassay as previously described [26].

### Immunohistochemistry

Deeply anesthetized rats were transcardially perfused with 150 ml of 0.9% heparinized saline at room temperature followed by perfusion with 500 ml ice-cold 4% paraformaldehyde (PFA) in 0.1M PBS. The brains were removed and post-fixed in 4% PFA for 24 hours. Subsequently, the brains were transferred to 30% sucrose until they sank and were then cut in 25 µm sections using a cryostat, thaw mounted on Superfrost plus slides (3 sections/slide) and air dried for 10 minutes before incubation in blocking buffer containing 0.3% Triton X-100 (in 10 mM Tris-phosphate buffered saline) and 5% normal horse serum. Subsequently, sections were incubated overnight at 4°C with primary polyclonal rabbit anti-CRF (1∶5,000, a kind gift of Dr. Wylie Vale) in buffer containing 0.3% Triton-X-100 and 1% normal horse serum. The next morning, slides were washed in PBS (3x, 5 min each), and incubated with a Cy3-conjugated affinity purified donkey anti-rabbit secondary antibody diluted 1∶500 in the same buffer as used for dilution primary antibody, for 2 hours at room temperature. Sections were washed 3x in PBS for 5 min and cover-slipped with Vectashield containing DAPI. Sections were visualized using Nikon fluorescent microscopy and images were acquired using a Zeiss Axiocam camera. A minimum of three sections per rat and 4 rats per group were analyzed. Pixel densities were quantified using Image J program (NIH). Anatomical boundaries of specific nuclei and brain regions were verified using the Rat Brain atlas by Paxinos and Watson [27].

### Functional ablation of gastric afferent nerves by intragastric resiniferatoxin (RTX) and measurement of gastric splanchnic nerve (GSN) activity

The capsaicin analog, RTX, reliably produces a robust and long-lasting (weeks to months) chemical de-afferentation [28] and is an ultrapotent TRPV1 receptor agonist that causes desensitization of extrinsic afferents when applied locally, was used to assess the effects of ongoing activity in these nerves on depression-like behavior. In this study RTX was instilled in the stomach to desensitize gastric afferent nerves. RTX was dissolved in a mixture of 10% ethanol +10% Tween-80 in 0.9% saline in the ratio of 1∶1∶8. After laparotomy, the stomach was exposed and the pylorus was clamped. RTX (25 ug/kg in 0.8 ml saline) or vehicle (n = 8 for each group) was administered directly into the gastric lumen with a thin needle attached to a glass syringe. After 30 minutes the RTX solution in the stomach was aspirated and the stomach was unclamped. The incision was then closed and rats were allowed to recover for a week before being subjected to the FST.

The sensory effect of RTX was verified in a separate group of rats in whom GSN activity was measured as previously described [11]. Briefly, rats were anesthetized with sodium pentobarbital (50 mg/kg, intraperitoneally) and maintained with a constant infusion of 50 mg sodium pentobarbital in 9 ml, 0.9% NaCl at 1.0 ml/h through the jugular vein. Tracheotomy was performed for artificial ventilation and rats were paralyzed with gallamine triethiodide 20 mg/kg. i.v; Sigma). A balloon (2.5 cm long) attached to a catheter (PE-240) was placed in the stomach through a small incision. The left GSN was exposed just below the diaphragm and the distal segment was placed in a pool of warm mineral oil and teased into fine bundles and nerve activity recorded by a bipolar silver hook electrode. Action potentials were amplified by a low-noise AC differential amplification (Iso-DAM8A Bio-amplifier, WPI) with 300 Hz-10 Khz filter, processed and analyzed through using the CED 1401/SPIKE 2 program (Cambridge Electronic Design, UK). Action potentials responsive to gastric distention (GD) were identified. Intragastric pressure (80 mmHg) was produced by rapidly inflating the balloon for the duration of 20 seconds via a pressure transducer and sphygmomanometer. Single unit recordings were differentiated and compiled into rate histograms (1 second bin width) using wave-mark template in SPIKE 2. RTX or vehicle (n = 4 for each group) was administered into stomach as previously described. The GSN response to RTX or vehicle at baseline and in response to GD (80 mmHg) was recorded at baseline and 90 minutes after treatment.

### Antalarmin administration

In a separate group of rats, the CRF1 receptor antagonist, antalarmin (Sigma-Aldrich, St. Louis, MO) was dissolved in distilled water containing 0.1% Tween 80. Antalarmin (10 mg/kg) or vehicle was administered by oral gavage in a volume of 5 ml/kg (n = 6 for each treatment group). Each rat received two injections of antalarmin, 15 min after the training of FST on day 1 and 60 min before FST test on day 2. [29].

### Data Analysis

All values were presented as means ± SE. Student's t-test was used to analyze the results of the FST, EPM, OFT, LDB, immunohistochemistry and gastric splanchnic nerve activity. The sucrose preference test was analyzed by Chi-square test and Student's t-test. The HPA response (ACTH and corticosterone) was analyzed by two-way ANOVA and Student- Newman Keuls test. Statistical analysis was performed using StatView (Abacus Concepts Inc, Berkeley, CA).

## Results

### Behavioral Testing for Depression-like Behavior

#### Sucrose Preference Test

**Figure 1:**
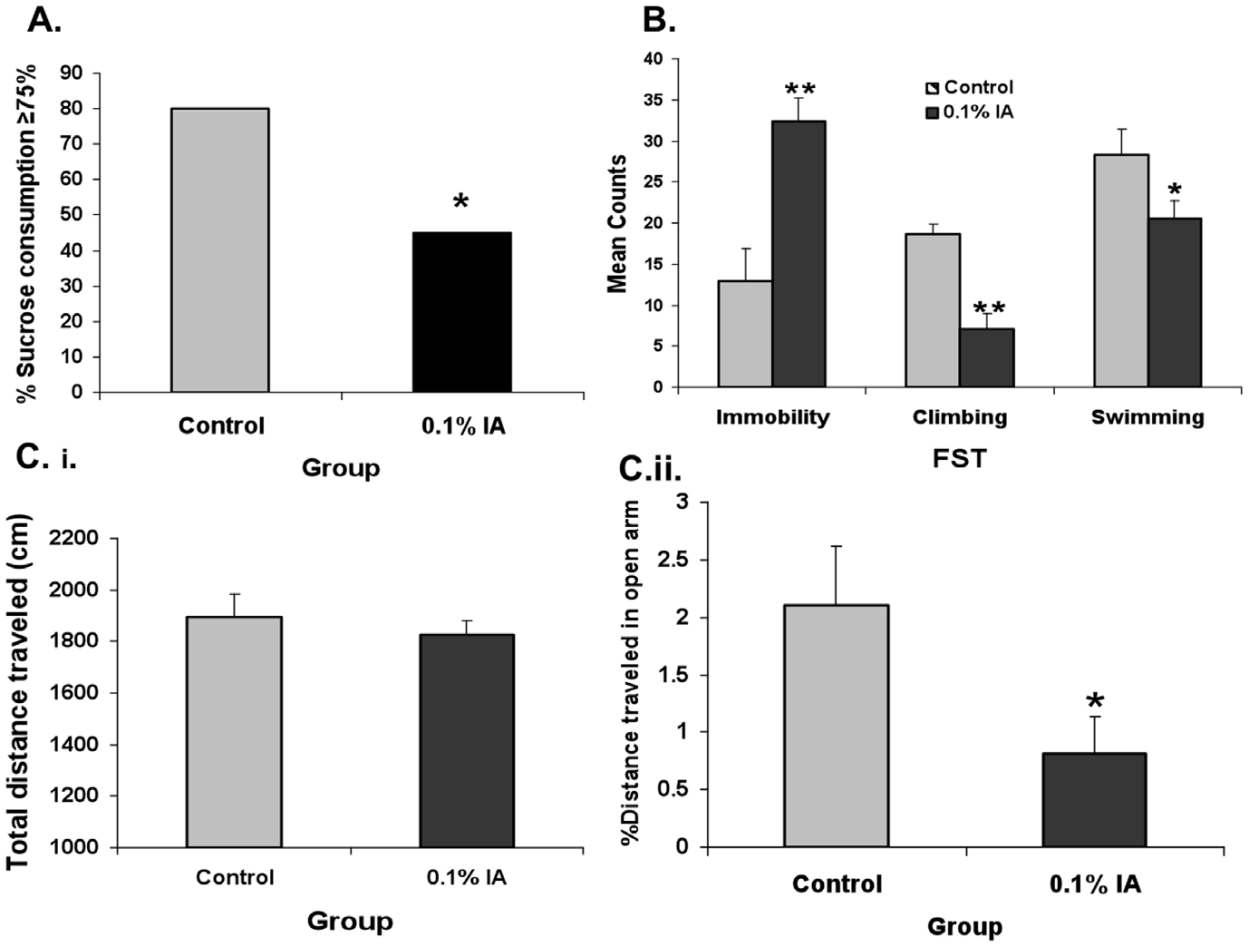
The effect of neonatal IA treatment on psychological behavior of adult rats. **A.** ***Sucrose consumption test***. The percentage of animals with sucrose consumption ≥75% was significantly lower in IA-treated rats as compared with control rats (*P<0.05) (see text for details). **B.** ***Forced swimming test***. Immobility was significantly increased (P<0.001) and the climbing and swimming were significantly reduced (P<0.001 and P<0.05) in IA-treated rats compared with control rats (see text for details). Data represent the mean ± SEM of 20 rats per group. **C.** ***Elevated plus maze test***. **i.** The total distance traveled (cm) in the 5-minute test time was not different between the IA-treated and control groups. **ii.** The percentage distance traveled in open arm was significantly reduced in IA-treated rats.

The sucrose and water intake in IA-treated rats was not significantly different as compared with control rats (7.95±0.70 and 3.20±0.37 g vs. 9.20±0.73 and 2.40±0.27 g, respectively; P>0.05). However, the percentage of rats with sucrose consumption of ≥75% was significantly reduced in IA-treated rats (45%) relative to the control rats (80%, P<0.05 by Chi-square test, n = 20 per group; Figure 1A).

#### Forced Swimming Test

Compared with controls, immobility, as measured by the number of 5-second intervals with no motion at the end, was significantly increased in IA-treated rats during the 5-minute test time (32.40±2.83 vs. 12.95±4.0; P<0.001, n = 20 in each group), whereas the number of climbing and swimming behaviors were reduced (7.05±1.18 vs. 18.65±1.90; P<0.001 and 20.6±3.02 vs. 28.35±2.09 respectively; P<0.05, Figure 1 B).

### Behavioral Testing for Anxiety-like Behavior

#### Elevated Plus Maze Test

The total distance traveled during the 5-minute test time was not significantly different between IA-treated and control rats (1823.86±58.07 vs. 1893.12±92.24 cm; P = 0.5, n = 20/group, Figure 1 C.i.). Similarly, the number of entries into the open arm was also not significantly different between IA-treated or control rats (1.95±0.67 vs. 2.75±0.56, P = 0.362) suggesting that the locomotor activity was not altered in IA-treated rats. However, the percentage of the distance traveled on the open arms was significantly reduced in IA-treated rats compared with control (0.81±0.31% vs. 2.1±0.52%; P = 0.05, Figure 1 C.ii.) with the percentage of time spent on the open arms being similar in both groups (1.42±0.48% vs. 2.34±0.47%; P = 0.18).

#### Open Field Test

**Figure 2:**
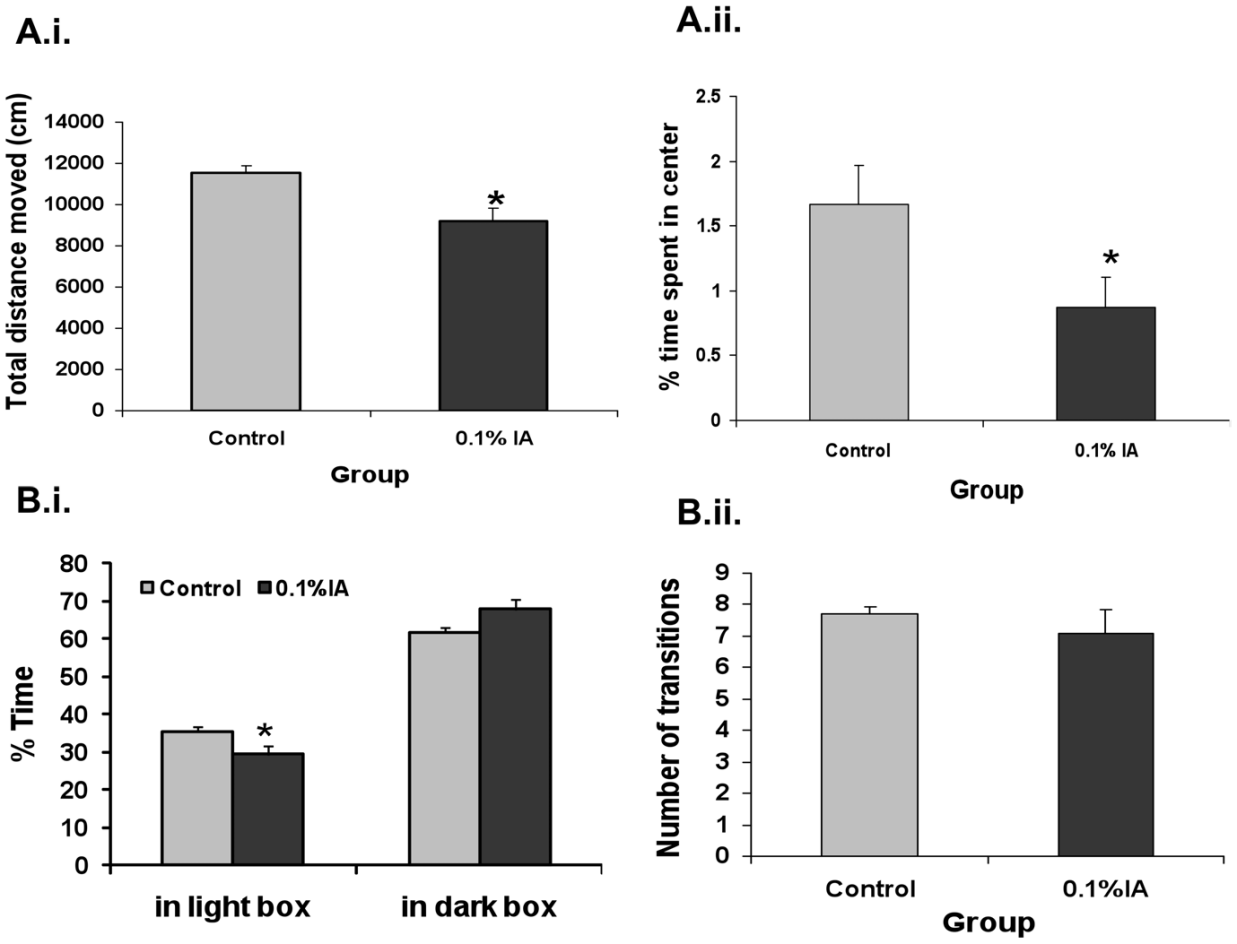
The effect of neonatal IA treatment on psychological behavior of adult rats (continued). **A.** ***Open field test***. **i.** The total distance traveled (cm) in the 30-minute test time. **ii.** The percentage time spent in center. There was a significant decrease in the time that IA-treated rats spent in the center as well as the total distance traveled. Data represent the mean ± SEM of 20 rats per group. **B.** ***Light dark box test***. **i.** The percent of time spent in the light box was significantly shorter in the IA-treated rats as compared with control rats. **ii.** The number of light-dark box transitions was not significantly different between the two groups (n = 10 per group). (* =  Significantly different from control group).

The total distance traveled during the 30-minute test was significantly reduced in IA-treated rats relative to control rats (P<0.05, n = 20 in each group, Figure 2A.i.), suggesting a reduction in locomotor activity. Although the distance traveled in the center was not significantly different between IA-treated and control rats (236.37±56.99 vs. 345.84±62.66 cm; P = 0.20), the percentage of time spent in the center was shorter in IA-treated rats as compared with control rats (0.87±0.23%vs. 1.67±0.31%; P<0.05, Figure 2A.ii.).

#### Light Dark Box Test

The percentage time spent in the light box was significantly shorter in IA-treated rats than that in control rats (29.39±2.54% vs 35.61±1.33%.; P<0.05, n = 10 for each group, Figure 2 B.i.) although the number of light-dark box transitions was similar in the IA-treated and control rats (7.10±0.72 vs. 7.70±0.21; P>0.05, Figure 2 B.ii.).

### Hormonal responses to minor stress

**Figure 3:**
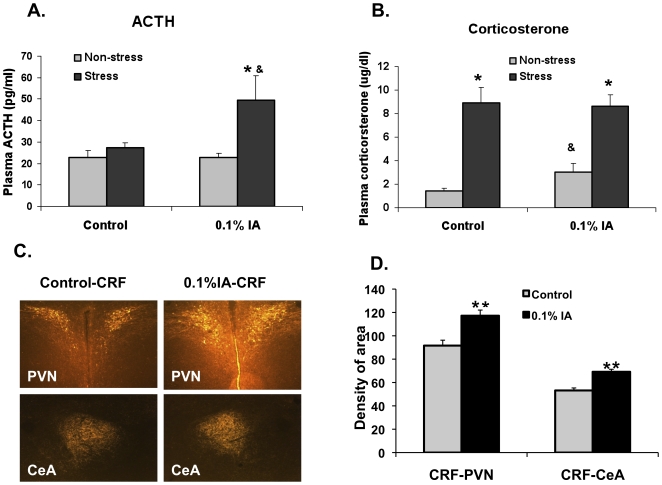
ACTH and corticosterone responses to minor stress (saline injection). **A.** Saline injection significantly increased ACTH secretion in IA-treated rats (* P<0.05 compared with stress control rats; **&** P<0.05 compared with non-stress IA-treated rats). **B.** The basal level of corticosterone was increased in IA treated rats than control rats (**&** P<0.05 compared with non-stress control rats; * P<0.05 stress rats compared with non-stress rats) (see text for details). Data represent the mean ± SEM of 8 rats per group. (* =  Significantly different from control group) **C.** Representative Photographs (Magnification: 10x) of CRF change in paraventricular nucleus (PVN) and central nucleus of the amgdyla (CeA). **D.** Change of CRF staining Density in PVN and CeA area. Data represent the mean ± SEM of 3 sections per rat, 4 rats per group. (* =  Significantly different from control group).

To determine the sensitivity to stress, we measured plasma ACTH and corticosterone levels 15 minutes after a minor stress in the form of saline injection. In control rats, saline injection did not significantly alter plasma ACTH concentration but did increase corticosterone levels (Figure 3A and 3B, n = 8 in each group). In the IA-treated rats, saline injection significantly increased both ACTH and corticosterone levels secretion. Furthermore, the basal level of corticosterone was increased in IA treated rats (3.05±0.74 vs. 1.41±0.25 µg/dl; P<0.05, n = 8 in each group), suggesting an ongoing stress response as well as increased sensitivity to stress.

### Hypothalamic CRF expression

As compared with controls, CRF immunoreactivity was increased in IA-treated rats in both the hypothalamic paraventricular nucleus (PVN) (IA-treated versus controls: 117.19±4.83 vs. 91.60±4.63; P<0.001; arbitrary units ± SEM) and the central nucleus of the amygdala (CeA) (69.12±2.17 vs. 53.19±2.14 arbitrary units; P<0.001, n = 4 in each group) (Fig. 3C and 3D).

### Effects of gastric afferent ablation on depression-like behavior

**Figure 4:**
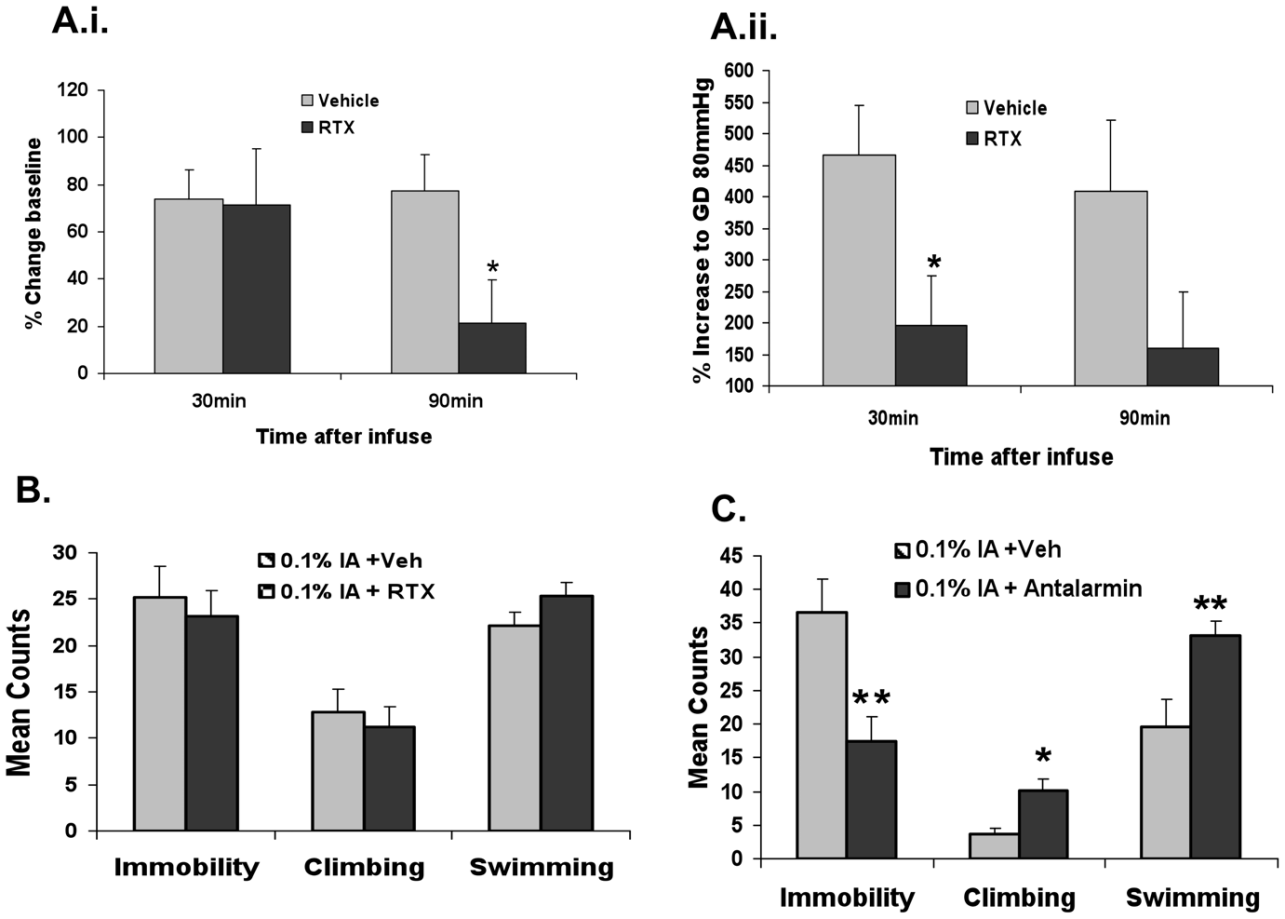
Effects of intragastric RTX and antalarmin. **A: Gastric afferent activity after intragastric RTX as compared to vehicle treatement.** **A.i:** There is a significant (p<0.05 using ANOVA) decline in basal splanchnic nerve activity after RTX but not in vehicle treated group. **A.ii:** There is a significant (p<0.05 using ANOVA) decline in the evoked response to gastric distention, GD, at 80 mmHg of pressure) 30 minutes after RTX but no change in the vehicle treated group (n =  4 each). (*  =  Significantly different than Pre-treatment baseline using Bunnet multiple comparison test). **B: Effects of gastric sensory ablation on depression-like behavior.** No differences were seen in the results of the forced swimming test in IA-treated animals that underwent intragastric RTX treatment as compared with animals that received vehicle only. **C. Effects of the CRF1 antagonist antalarmin on depression-like behavior.** Antalarmin resulted in a significant reduced in immobility and an increase in the climbing and swimming in IA-treated rats compared with vehicle treated IA-treated rats.

In short-term studies, we confirmed that RTX-, but not vehicle, treatment effectively abolished spinal nerve basal activity (Figure 4A. i., n = 4 in each group) and response to gastric distention (Figure 4A.ii.). RTX reliably produces a robust and long-lasting (weeks to months) chemical de-afferentation [28]. Because we saw a more profound effect of FD on depression-, but not anxiety-like behavior, we next tested the effect of RTX treatment on behavior. Seven days after RTX treatment, the forced swimming test was performed and showed that depression-like behavior was unaffected by RTX treatment (Figure 34B, n = 8 in in each group).

### Effects of the CRF antagonist antalarmin on depression-like behavior

In IA-treated rats, antalarmin significantly decreased immobility, as measured by the number of 5-second intervals with no motion (17.5±3.53 vs. 36.67±4.81, P<0.001) and increased numbers of swimming (33.17±2.2 vs. 19.67±4.06, P<0.05) as well as climbing behavior (10.17±1.6 vs. 3.67±0.95, P<0.001) (n = 6 in each group, Fig. 4C).

## Discussion

A recent study has suggested that symptom severity in patients with functional dyspepsia is strongly associated with psychosocial factors (depression, abuse history) and somatization, and only to a lesser extent by gastric sensorimotor function [5]. However, psychological abnormalities could either be driving abdominal symptoms or conversely, could be a result of gastrointestinal abnormalities. Answering this question in humans may be difficult because of the need for longitudinal studies that document the onset of psychosocial dysfunction in relationship to visceral symptoms [30]. On the other hand, a causal relationship is easier to explore in experimental models. In this study, we therefore used a previously validated rat model of functional dyspepsia, induced by early life gastric irritation, to examine the relationship between FD and psychological manifestations. We show that transient gastric irritation in the neonatal period can result in long lasting depression-like behavior, impaired HPA axis and upregulation of hypothalamic CRF. Taken together with our previously published data on gastric hypersensitivity and impaired accommodation [11] these results therefore provide an alternative explanation for the strong association between functional disorders and the well-described neuropsychiatric manifestations[31]-[34].

We used several tests to evaluate depression-like and anxiety-like behaviors experimental rats. Decrease of sucrose solution consumption reflects anhedonia (decreased ability to experience pleasures), a core symptom of depressive disorder [16]. FST was originally used for screening antidepressants that decrease the immobility and the “learned helplessness” of this test can be used as a surrogate for depression, although it may be better to regard this a measure of the ability to cope with stress [18], [19]. In the OFT, the time spent in the center of the field correlates inversely with anxiety-like behavior [22], [23]. In this study, IA-treated rats spent less time in the center of the open field as compared with control rats. The elevated plus-maze (EPM) is widely used test to measure fear or anxiety-like behaviors in response to a novel environment and height [21]. The time spent in the open arms correlates inversely with an anxiety-like state. In the present study, although the time spent in the open arm was not significantly different between the two groups, the percentage of the distance traveled in the open arm of EPM was significantly reduced in the IA-treated group as compared with control rats. The light dark box (LDB) test also is used for testing of anxiety-like behaviors [24], [25]. In this study, although there is no difference in light-dark transitions between the two groups, IA-treated rats spent significantly less time in the light box. It has been argued that time spent in the light box is a more accurate measure of anxiety in rodents than the frequency of transition, which may be more related to locomotor activity [35]. Taken together, therefore, our results suggest that neonatally sensitized adult rats display significantly increased depression-like and to a lesser extent, anxiety-like behaviors, both hallmarks of humans with FD.

We have previously shown that neonatal rats treated with IA have gastric sensitization and increased gastric spinal afferent activity and therefore it was possible that these rats are in chronic pain, which in turn was contributing to depression- and anxiety-like behaviors, an association that has been well described in the literature [36]–[39]. We eliminated this possibility by using intragastric RTX to abolish spinal afferent signaling and showed that this had no effect on depression-like behavior, suggesting that the observed effects reflected a permanent change in the nervous system in response to the neonatal irritation.

Phenotypic plasticity in response to environmental changes is a hallmark of living organisms and is a critical biological process determining health and disease in adult animals. An equally profound effect on the future health of the organism may result from such changes in early life, either in the pre-gestational or neonatal period. Our results reinforce this growing but important paradigm in medicine and biology, known as the developmental origin of health and diseases (DOHaD), an extension of the original Barker's hypothesis [40]. This plasticity or “programming” is an adaptive response to a variety of environmental stressors occurring at a uniquely vulnerable period in the lifespan of the living being. These studies are in keeping with previous studies from our laboratory, showing that long lasting visceral hypersensitivity can result from transient irritation of the colon or stomach in the neonatal period [11], [12]. The present study now extends this concept to the realm of functional gastrointestinal disorders by showing how transient gastric irritation in the neonatal period can result in significant changes in psychological behavior.

In recent years, experimental studies have begun to provide insight into the biological basis of this form of neonatal programming with a focus on the hypothalamic–pituitary–adrenal (HPA) axis, involving the release of CRF from the parvocellular neurons in the hypothalamic paraventricular nucleus [41]. We therefore examined the effects of transient neonatal gastric inflammation on CRF expression and the HPA axis response to stress. The expression of CRF in the hypothalamus was significantly increased in IA-treated rats as compared with controls. In response to saline injection (a minor stressor) [42], a robust increase in ACTH was seen in IA-treated rats, whereas no significant change was observed in the control rats. Along with the elevation of baseline corticosterone levels, our results suggest overall that the HPA axis is impaired in IA-treated rats. Finally, we used antalarmin, a potent antagonist of the CRF1 receptor that has previously been shown not to affect the results of FST in normal rats [43], [44]. The fact that it reverses depression-like behavior in IA-treated rats in our study, along with elevated levels of CRF expression, suggests a role for this peptide in the pathogenesis of the behavioral changes in our model. CRF1 plays a pivotal role in the stress response system, modulating behavior through multiple circuits in different regions of the brain including those responsible for arousal and coping and CRF1 antagonists including antalarmin have been useful in several preclinical models of depression [44], [45].

Our experimental results have human correlates with studies that support the neonatal insult theory for functional bowel disorders. There is substantial literature correlating visceral symptoms and psychosocial dysfunction in patients with IBS and early life trauma [46]–[49]. More direct evidence also comes from a clinical case-control study using the Swedish National Registry showing that gastric suction at birth may promote the development of long-term visceral hypersensitivity and cognitive hypervigilance in adults [50]. It has also been suggested that humans who experience an early adverse life event before the age of 14 have a hyper-responsive HPA and that HPA reactivity may in turn modulate IBS symptoms [48].

Further studies will be required to understand the mechanisms sustaining the upregulation of CRF such as impaired feedback signaling from epigenetic changes regulating glucocorticoid receptor expression as has been reported in other forms of environmental reprogramming [41]. What initiates this cascade of events in the neonatal period is also unknown. Simply handling neonates may result in anxiety-like behaviors and gene plasticity [51]–[55] but this is unlikely to have contributed to our findings since both control and IA-treated rats were handled similarly. Transient gastritis may alter the HPA axis through activation of cytokines, as has been reported in patients with irritable bowel syndrome [46]. Gastric afferent nerves may also play a role in the initiation of the behavioral effects. In addition to spinal nerve activity along nociceptive pathways, intragastric acid can also activate sub-cortical brain nuclei (including the CeA and anterior of part of the PVN) via vagal pathways [56].

In conclusion, our results demonstrate, for the first time, that transient gastric irritation in the neonatal period can induce long-lasting depression-like and anxiety-like behaviors that are accompanied by increased central CRF expression and a dysregulated HPA axis and are mediated by CRF1 receptors. These findings have major implications for the pathogenesis of psychological co-morbidity in patients with FD: early life gastrointestinal events not only result in altered visceral sensorimotor function, but can also induce a phenotype of depression and anxiety in adult life, thus offering a theoretically satisfying explanation for the strong association between the two phenomena.
